# First description of phosphofructokinase deficiency in spain: identification of a novel homozygous missense mutation in the PFKM gene

**DOI:** 10.3389/fphys.2013.00393

**Published:** 2013-12-30

**Authors:** Joan-Lluis Vives-Corrons, Pavla Koralkova, Josep M. Grau, Maria del Mar Mañú Pereira, Richard Van Wijk

**Affiliations:** ^1^Red Cell Pathology Unit, Biomedical Dianostic Centre, University Hospital Clínic de BarcelonaBarcelona, Spain; ^2^Faculty of Medicine and Dentistry, Department of Biology, Palacky UniversityOlomouc, Czech Republic; ^3^Service of Internal Medicine and Muscle, University Hospital Clínic de BarcelonaBarcelona, Spain; ^4^Department of Clinical Chemistry and Haematology, University Medical Center UtrechtUtrecht, Netherlands

**Keywords:** phosphofructokinase deficiency, glycogen storage disease, PFKM gene, missense mutation, enzyme catalysis

## Abstract

Phosphofructokinase deficiency is a very rare autosomal recessive disorder, which belongs to group of rare inborn errors of metabolism called glycogen storage disease. Here we report on a new mutation in the phosphofructokinase (PFK) gene *PFKM* identified in a 65-years-old woman who suffered from lifelong intermittent muscle weakness and painful spasms of random occurrence, episodic dark urines, and slight haemolytic anemia. After ruling out the most common causes of chronic haemolytic anemia, the study of a panel of 24 enzyme activities showed a markedly decreased PFK activity in red blood cells (RBCs) from the patient. DNA sequence analysis of the *PFKM* gene subsequently revealed a novel homozygous mutation: c.926A>G; p.Asp309Gly. This mutation is predicted to severely affect enzyme catalysis thereby accounting for the observed enzyme deficiency. This case represents a prime example of classical PFK deficiency and is the first reported case of this very rare red blood cell disorder in Spain.

## Introduction

**Figure 1:**
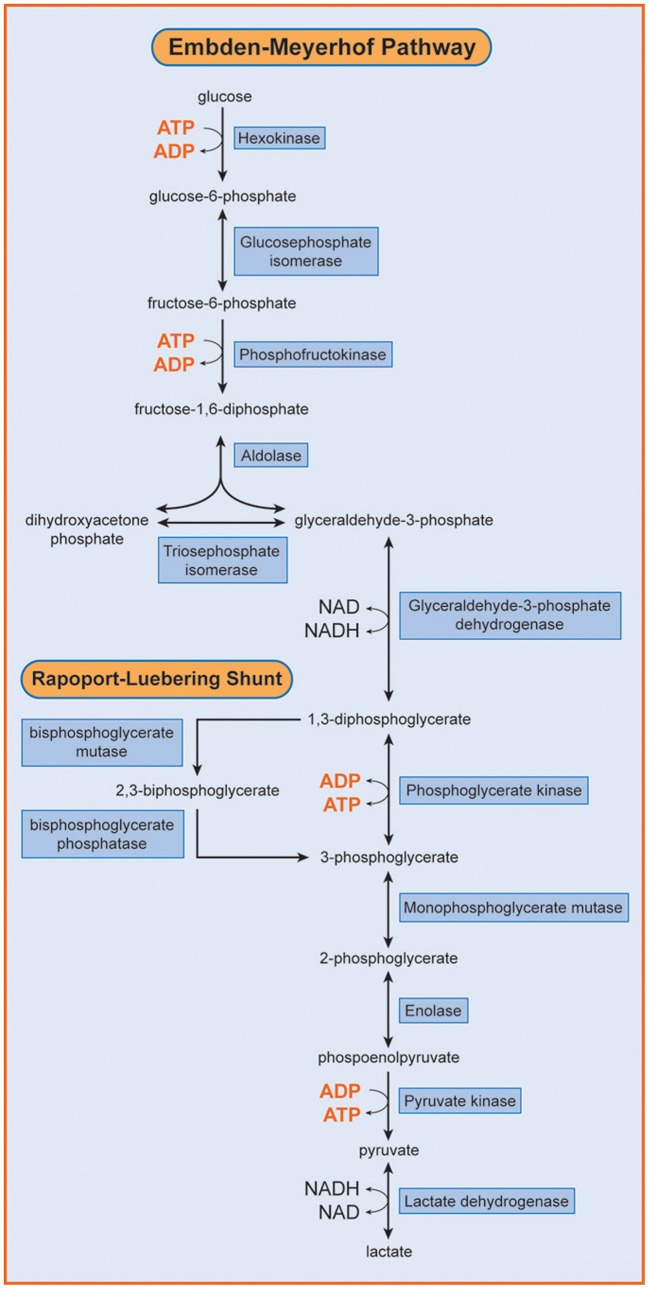
**Embden Meyerhof Pathway of RBC metabolism**. Phosphofructokinase (PFK) catalyzes the transformation of fructose 6-phosphate into fructose 1,6 diphosphate. [Reproduced with permission from Van Wijk and van Solinge (2005)]

Phosphofructokinase (ATP: D- fructose-6-phosphate-1-phosphotransferase; EC 2.7.1.11; PFK) is a key regulatory enzyme of the glycolytic cycle and catalyses the conversion of fructose-6-phosphate to fructose-1,6-diphosphate (Figure 1). Human PFK is composed of three isoenzymes, muscle (M), liver (L), and platelet (P) (Vora, 1983; Nakajima et al., 2002). The P type is also known as Fibroblast type (F). Mammalian PFK is a tetrameric enzyme that is subjected to allosteric regulation. Tissue isozymes randomly aggregate to form homotetramers or heterotetramers depending on the relative abundance of the subunits in a particular tissue. PFK-M is the sole subunit in muscle cells whereas red blood cells (RBCs) contain both L and M subunits and form their hybrids (M4, L4, M3L, M2L2, and ML3).

Phosphofructokinase deficiency (OMIM 171 850) is a very rare autosomal recessive condition with heterogeneous clinical symptoms, mainly characterized by myopathy and/or haemolysis (Hirano and Di Mauro, 1999). Myopathy is caused by the accumulation of glycogen in muscle tissue due to the metabolic defect and is also known as glycogenosis type VII or Tarui disease. It is characterized by muscle pain, exercise-induced fatigue, cramps, and myoglobinuria. The observed clinical symptoms reflect lack of muscle PFK activity and partial reduction of enzymatic activity in erythrocytes. The latter usually is associated with mild haemolysis.

Up to now, only about 100 patients with PFK deficiency have been reported worldwide and 22 PFK-deficient *PFKM* alleles have been characterized. The gene encoding the M subunit (*PFKM*) has been assigned to chromosome 12q13.3 and spans 30 kb. It contains 24 exons and at least 3 promoter regions (Elson et al., 1990; Yamada et al., 2004). Among the detected mutations are mostly missense mutations and splicing defects.

We now describe here a Spanish patient with a clinical history of anemia, haemolysis, and intermittent muscle weakness, who was found to be homozygous for a novel mutation in the *PFKM* gene (c.926A>G). This mutation encodes the substitution of aspartic acid by glycine at residue 309 (p.Asp309Gly). This is the first description of PFK deficiency in Spain.

## Case report

A 65-years-old woman with long standing hypertension and type 2 diabetes was referred to our Unit because of intolerance to exercise and chronic fatigue. From youth she suffered from spasms of random occurrence associated with muscle weakness, painful intolerance to small efforts, and intermittent dark urines, especially after exercise due to myoglobinuria, as revealed by urinalysis. Physical examination showed no weakness or muscle atrophy and only a moderate splenomegaly without hepatomegaly or lymphadenopathy. Parents were unavailable for study and there was no known consanguinity. The same clinical picture, however, was present in a non-smoker brother whereas the three patient's daughters were normal. Patient's clinical condition has remained stable in follow-up.

Complete blood count (CBC) showed moderate anemia (Hb: 115 g/L), with slight macrocytosis (105 fl) and increased number of circulating reticulocytes (110 × 10^9^/L). Leukocyte and platelet counts, as well as general serum biochemical analysis, were within normal range, except for a moderate rise in non-conjugated bilirubin, lactate dehydrogenase (LDH) and uric acid (hyperuricemia). Biological signs of diabetes mellitus type 2 were also present. The studies performed to rule out the origin of the anemia, discarded nutritional deficiencies (serum iron tests, cobalamin and serum folate were all normal), haemoglobinopathies (HPLC and thermal stability), and paroxysmal nocturnal haemoglobinuria (normal flow cytometry measurement of CD45 and CD49 in leukocytes and RBCs). Hereditary RBC membrane defects were ruled out by morphological observation of May-Grünwald-Giemas stained blood smears, and a normal osmotic fragility test. Extensive study of RBC enzyme activity measurements demonstrated a marked decrease (<30% of normal) in PFK activity (Table 1). DNA sequence analysis of individual exons of *PFKM*, including flanking splice sites, revealed that the patient was homozygous for a missense mutation in exon 11: c.929A>G. This mutation, that has not been previously reported in the literature, encodes the substitution of aspartic acid by glycine at residue 309 (p.Asp309Gly). Mutation prediction programs PolyPhen-2 (Adzhubei et al., 2010) and SIFT (Kumar et al., 2009) predict this mutation to be pathogenic (i.e., disease causing).

**Table 1:** **Red blood cell enzyme activity measurements**.

	**Patient**	**Reference value**
G6PD activity (IU/g Hb)	7.0	6.2–9.9
HK activity (IU/g Hb)	1.2	0.6–1.3
PGI activity (IU/g Hb)	61.9	46.0–66.0
PK activity (IU/g Hb)	15.3	9.5–15.6
PFK activity (IU/g Hb)	3.07	6.4–13.9

Glucose-6-phosphate dehydrogenase; HK, Hexokinase; PGI, Phosphoglucose isomerase; PK, Pyruvate kinase; PFK, Phosphofructokinase.

The complete lack of PFK activity in muscle was confirmed on both histological preparations and muscle extracts. Muscle abnormality was also confirmed by electromyography (EMG), that showed mild myopathic changes and by the forearm test, characterized by a plane lactate curve with normal increase of ammonium. As usual in patients with glycogenosis, a painful spasm occurred at the end of the forearm test. Muscle biopsy showed slight amounts of polysaccharide (PAS) not digested by diastase and abnormalities in NADH-TR reaction.

## Discussion

**Figure 2:**
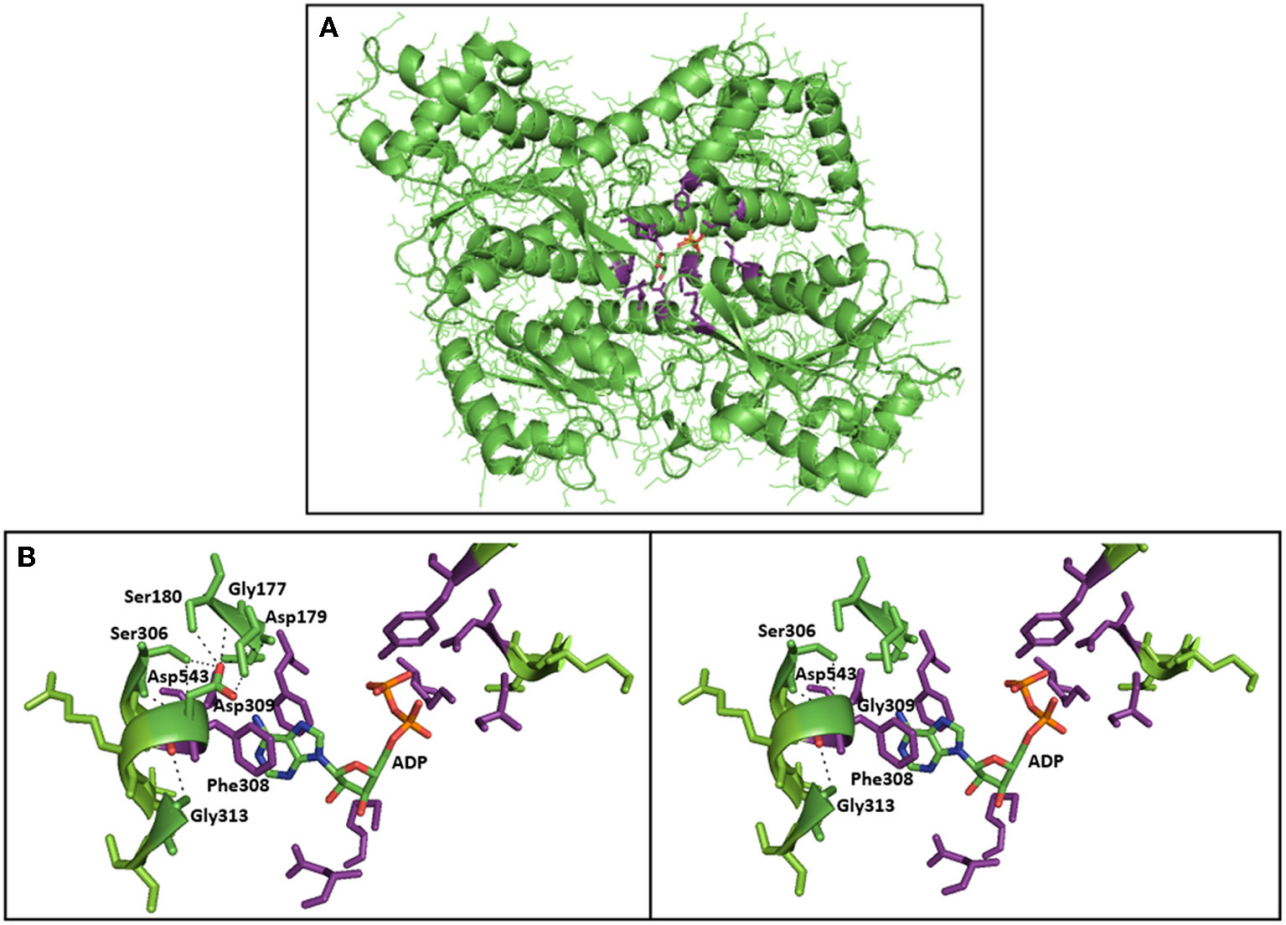
**3D crystal structure of PFK subunit from rabbit skeletal muscle and 3D model of normal/mutated PFKM (PDB 3O8L—protein databank). (A)** Allosteric nucleotide (ADP) binding site residues (purple) in the center of the rabbit subunit of PFKM, and the allosteric activator ADP are shown (atom coloring: carbon—green, nitrogen—blue, oxygen—red, phosphor—orange). **(B)** Close-up of the c.Asp309Gly mutation in PFKM. Asp309 is in close proximity of the ADP binding site. Asp309 does not directly interact with ADP. However, substitution of Asp309 by glycine is likely to disrupt multiple hydrogen bonds with Gly177, Gly179, Ser 180 and Ser306 (black dotted lines). This loss of hydrogen bonds, and the change of polarity and electrostatic interactions upon mutation of Asp309 will likely affect correct positioning of the side-chains of amino acids directly involved in ADP binding; in particular neighboring residue Phe308, which makes stacking interaction with the adenine ring of ADP.

In this report, we describe, for the first time, the occurrence of PFK deficiency in Spain in an adult woman of 65 years of age. Her moderate haemolysis was associated with the accumulation of muscle glycogen comparable to that of Tarui disease. Muscle pain and exercise-induced fatigue and weakness associated with dark urines were the most relevant clinical manifestations. She was found to be homozygous for a novel missense mutation in *PFKM*, the gene that encodes the PFK-M subunit. On the amino acid level, this mutation (c.929A>G) causes the substitution of aspartic acid by glycine at residue 309. Asp309 is part of an α-helix, and replacement with glycine could disrupt this α-helix. Furthermore, structural analysis using the 3D molecular model of rabbit muscle type PFK (PDB entry 3O8L) showed that the p.Asp309Gly substitution is located in direct vicinity of the nucleotide (ADP) binding site in the center of the PFKM subunit (Figure 2A). PFK is allosterically activated by ADP, and the ADP binding site has been recently identified (Banaszak et al., 2011). Asp309 does not directly interact with ADP, however, substitution of Asp309 by glycine is likely to disrupt multiple hydrogen bonds with Gly177, Gly179, Ser 180, and Ser306 (Figure 2B). This loss of hydrogen bonds and the change of polarity and electrostatic interactions upon mutation of Asp309 will likely affect correct positioning of the side-chains of amino acids directly involved in ADP binding (Figure 2B), in particular neighboring residue Phe308, which makes stacking interaction with the adenine ring of ADP. This hypothesis is further supported by recent functional studies of the Asp543Ala change in PFKM. This mutation is associated with Tarui disease, and substitution of Asp543 by Ala was shown to disrupt hydrogen bonds with ADP (Brüser et al., 2012). We therefore postulate that decreased binding of the allosteric activator ADP will inhibit PFK enzymatic activity, in particular under low energy levels. These findings support the physiological importance of Asp390 for the recently identified ADP binding site.

The missense mutation is thus predicted to lead to a less functional PFK-M subunit. In accordance with this, the complete lack of PFK activity in muscle was confirmed on both histological preparations and muscle extracts. The muscle abnormality was also confirmed by electromyography, ischemic exercise testing, histochemistry and electron microscopy. Furthermore, partial red blood cell PFK deficiency was reflected by the moderately decreased enzymatic activity in red blood cells, leading to mild haemolytic anemia

**Figure 3:**
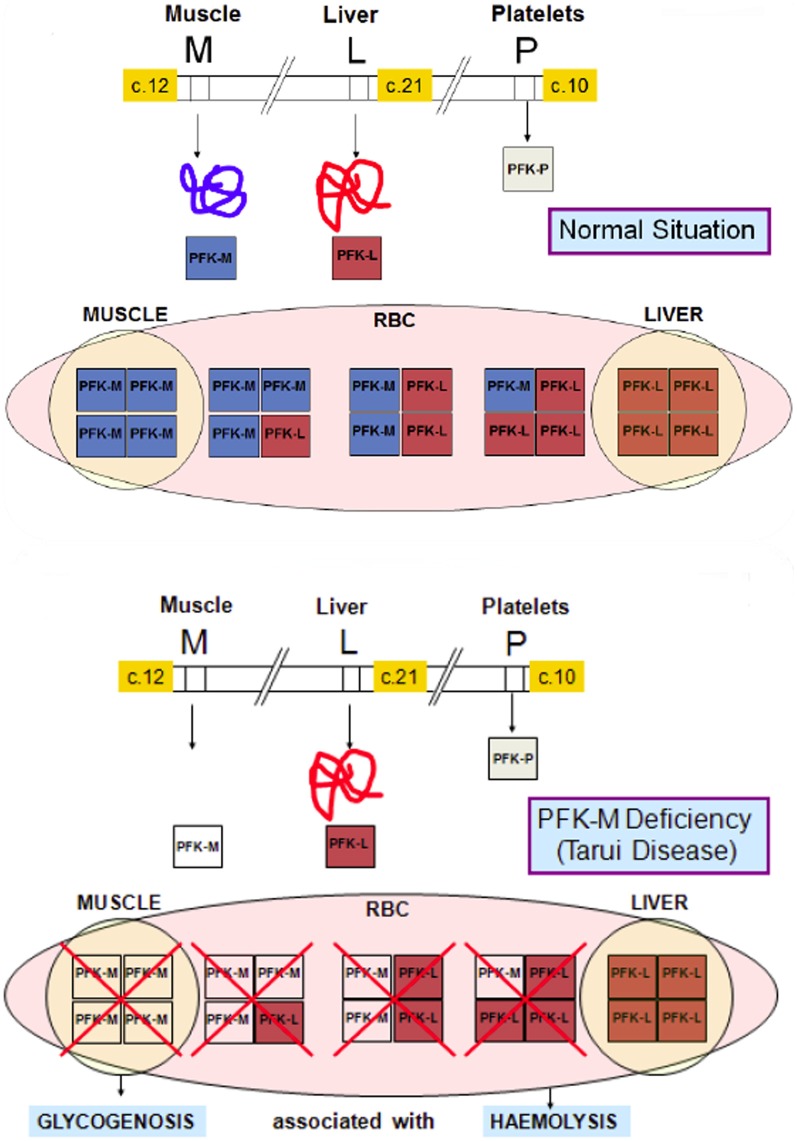
**Molecular basis of PFK deficiency**. In classic Tarui disease, the genetic defect involves the M isoform of PFK enzyme, resulting a severe enzyme deficiency in muscle. Erythrocytes that normally have two homotetramers (M4 and L4) and three hybrid isozymes (M3L, M2L2 and ML3), lack the M4 isoform and the hybrid isozymes, and only express the L4 homotetramers, resulting in about 50% of normal PFK activity

PFK is a key regulatory enzyme for glycolysis (Van Wijk and van Solinge, 2005) and catalyzes the irreversible transfer of phosphoryl from ATP to fructose-6-phosphate, and converts it to fructose-1,6-bisphosphate. Thus, tissues deficient in PFK cannot use free or glycogen-derived glucose as a fuel source and accumulate glycogen (glycogenosis). PFK deficiency (Tarui disease) was the first disorder recognized to directly affect glycolysis (Tarui et al., 1965). Since this first description of the disease, a wide range of biochemical, physiological and molecular studies have greatly contributed to our knowledge concerning not only PFK function in normal muscle, but also on the general control of glycolysis and glycogen metabolism. So far, more than one 100 patients have been described with prominent clinical symptoms characterized by muscle cramps, exercise intolerance, rhabdomyolysis and myoglobinuria, often associated with haemolytic anemia and hyperuricemia. In classic Tarui disease, the genetic defect involves the M isoform, resulting in the absence of enzymatic activity in the muscle. Erythrocytes lack the M4 and hybrid isozymes and only express the L4 homotetramers, resulting in about 50% of normal PFK activity (Figure 3). Thus, haemolysis is a result of partial erythrocyte PFK deficiency.

Despite PFK deficiency is a very rare autosomal recessive disease; its true incidence may be higher due to lack of recognition, as symptoms may be quite mild. In fact, our case highlights the latter, because the mild clinical presentation led her to be diagnosed, for many years, as chronic fatigue, until the consideration of dark urines ultimately led to the study of chronic haemolysis. In our case the combination of RBC enzyme activity measurements and muscle biopsy analysis allowed for the correct diagnosis even at this late stage of life.

Generally, PFK deficiency presents in childhood. Clinical history however, defines 4 main subtypes: (1) classic, (2) infantile onset, (3) late onset, and (4) haemolytic. Most of the reported cases belong to the classic form, characterized by exercise intolerance, fatigue, muscle cramps with pain and myoglobinuria (Hirano and Di Mauro, 1999). A compensated haemolysis with jaundice, increased serum creatine kinase (CK) and hyperuricemia is also commonly present. Sometimes, nausea and vomiting appear after intense physical efforts Patients with the infantile onset may manifest as “floppy babies” that die within the first year of life. Symptoms include myopathy, psychomotor retardation, cataracts, joint contractures, and death during early childhood. They can also show evidence of arthrogryposis and mental retardation. Patients with the late-onset form may present in adulthood with progressive muscle weakness, cramps and myalgias in later life. Exercise ability, however, is low already in childhood, and a mild muscle weakness may appear in the 5th decade leading to severe disability. Diagnosis depends on patient history, physical examination and the findings from muscle biopsy, electromyography, ischemic forearm testing, CK testing. RBC enzyme activity measurement is, however, the easiest way to establish the definitive diagnosis. Fatal infantile type and late-onset forms of PFK clinical expression are very rare, with only several reported cases. The haemolytic form presents with hereditary non-spherocytic haemolytic anemia without muscle manifestations (Fujii and Miwa, 2000). Due to the molecular genetic heterogeneity, a clear-cut genotype-phenotype correlation has not been recognized in patients with PFK deficiency (Toscano and Musumeci, 2007). Unfortunately, no specific treatment or cure of enzyme deficiency exists. Although diet therapy may be highly effective at reducing clinical manifestations, Tarui disease resolves with rest. Fortunately, the condition does not progress to severe disability. Because the liver and kidneys express only the L isoform, these organs are spared.

About 100 cases of PFK deficiency have been reported in patients with Tarui disease from Europe, USA, and Japan with some predominance in Ashkenazi Jews (Fujii and Miwa, 2000). Up to know, 22 different mutations of PFK have been described. Missense and splicing mutations are the most frequently occurring mutations in *PFKM*. Intriguingly, PFK-deficient Ashkenazi Jews share 2 common mutations in the gene. A splicing defect caused by the G>A base change at the first nucleotide in intron 5 (c.237+1G>A) accounts for 68% of mutant Ashkenazi alleles, and a single base deletion in exon 22 (c.2003delC) accounts for about 27% of mutant Ashkenazi alleles (Raben and Sherman, 1995). The here described homozygous patient is the first Spanish case described to be affected by this very rare disease. The identification of a novel homozygous missense mutation further extends the repertoire of PFK deficiency-associated mutations in *PFKM*.

### Conflict of interest statement

The authors declare that the research was conducted in the absence of any commercial or financial relationships that could be construed as a potential conflict of interest.
